# A Chinese expert consensus on thoracic endovascular aortic repair of type B aortic dissection with a single-branched stent graft for revascularization of the left subclavian artery

**DOI:** 10.3389/fsurg.2023.1230334

**Published:** 2023-08-17

**Authors:** Xiaoye Li, Chao Song, Lei Zhang, Liangxi Yuan, Xiangchen Dai, Lianrui Guo, Qingsheng Lu

**Affiliations:** ^1^ Department of Vascular Surgery, The First Affiliated Hospital of Naval Medical University, Shanghai, China; ^2^ Department of Vascular Surgery, General Hospital, Tianjin Medical University, Tianjin, China; ^3^ Department of Vascular Surgery, Xuan Wu Hospital, Capital Medical University, Beijing, China

**Keywords:** thoracic endovascular aortic repair, type B aortic dissection, left subclavian artery, consensus, branched stent graft

## Abstract

Thoracic endovascular repair (TEVAR) is currently the recommended and most widely used treatment for type B aortic dissection. A major challenge is revascularization of the left subclavian artery in order to extend the landing zone to zone 2 (Ishimaru classification). Various strategies have been used for revascularization, including branched stent graft, fenestrated stent graft, the chimney technique, the parallel technique, and bypass surgery. Single-branched stent graft is one of the most promising strategies, and several products have recently been reported as potential candidates for use with this approach. The Castor single-branched stent graft is the only off-the-shelf product available; this product has been developed through collaboration between Chinese corporations and clinicians. In this Perspective article, clinical experience and data obtained from TEVAR with the Castor single-branched stent graft are summarized by experienced Chinese experts.

## 1. Introduction

Aortic dissection is one of the most catastrophic diseases affecting the aorta. Without treatment, the mortality rate of acute aortic dissection will exceed 22.7% within 6 h, 50% within 24 h, and 68% within the first week (1). The management of type B aortic dissection (TBAD) has shifted over the last decade, and thoracic endovascular aortic repair (TEVAR) has become the recommended treatment for complicated hyperacute, acute, or subacute TBAD in cases of favorable anatomy (2, 3). The goal of TEVAR is to seal the proximal entry tear with a covered stent graft, thus inducing false lumen thrombosis, true lumen expansion, and restoration of blood flow.

In patients with TBAD, the primary entry tear typically originates a few centimeters distal to the ostium of the left subclavian artery (LSA) owing to the large fluctuation in dP/dt pressure at the curvature (4, 5). Msear et al. found that 89.5% of TBAD patients had less than 2 cm of healthy proximal descending thoracic aorta, which means a lack of sufficient proximal landing zone (6). Sufficient proximal landing zone, which is defined as the region between the proximal edge of the stent graft and the primary entry tear, is fundamental to the success of the procedure; hence, proximal extension of the stent graft is necessary. LSA coverage has been reported as one method of creating a sufficient landing zone; however, LSA coverage has subsequently been found to be associated with an increased risk of stroke and spinal cord ischemia (7, 8). Therefore, various strategies have been tested to revascularize the LSA in order to extend the stent graft to zone 2 [Ishimaru classification: from the distal edge of the ostium of the left common carotid artery (LCCA) to the distal edge of the ostium of the LSA], while preserving blood flow in the LSA. These strategies include branched stent graft, fenestrated stent graft, and parallel stent graft (9–11).

## 2. Branched stent graft for preservation of the LSA during TEVAR

Branched stent graft is one of the most commonly used strategies to preserve the LSA (the other being fenestrated stent graft). Several products have been reported as offering potential solutions for complete sealing in zone 2 while keeping the LSA patent; these include the Valiant Mona left subclavian artery (LSA) stent graft (Medtronic Inc., Santa Rosa, CA, USA) (12), the Gore Thoracic Branch Endoprosthesis (TBE, WL Gore, Flagstaff, AZ, USA) (13), and the Inoue Stent Graft (PTMC Institute, Kyoto, Japan) (14). The Castor single-branched stent graft (Microport Medical, Shanghai, China) has been approved by the Chinese Food and Drug Administration (CFDA) and has been made available as the first off-the-shelf single-branched stent graft. The initial indication for the Castor single-branched stent graft was TBAD, and this was later extended to cases of thoracic dilated aorta. To date, more than 800 experts from more than 700 centers have performed TEVAR with the Castor single-branched stent graft, and more than 1,000 patients have been successfully treated. Yao et al. performed a meta-analysis of TEVAR with the Castor single-branched stent graft for TBAD, which revealed that of a total of 415 patients, the LSA was successfully preserved in all cases. The 1-year overall survival rate was 99.7%, and the 1-year and 2-year LSA patency rates were 99.7% and 99.3%, respectively (15).

## 3. Castor single-branched stent-graft is specially designed for endovascular repair of TBAD

### 3.1. Unibody design with a flexible branch section

The Castor single-branched stent graft has a unibody design, which means that there is no gutter or space between the branch section and the main body. The unibody design of this stent graft could greatly reduce the risk of endoleak compared with the parallel stent graft (type Ia) and fenestrated stent graft (type II), and its multifilament PTE graft could also reduce the risk of type IV endoleak.

The ostium of the LSA can originate from various clock positions of the aortic arch. Yoon et al. found that the median clock position of the ostium of the LSA was 12:00 (range, 11:30–12:45), and Mougin et al. found that the median clock position of the ostium of the LSA relative to the ostium of the LCCA was −10 min (−30 to 0 min), suggesting low variability in the relative position of the LSA (16, 17). The branch section of the Castor single-branched stent graft can be rotated by up to 150 degrees in multiple directions while maintaining patency, which means that it can be fully adapted to different positions of the ostium of the LSA.

### 3.2. Patient selection

The Castor single-branched stent graft is specifically designed to align with the morphological characteristics of TBAD. According to the Instructions for Use (IFU), its indications include (1) thoracic aortic dissection retrograde to the LSA and (2) thoracic aortic dissection requiring proximal fixation in zone 2 and zone 3.

It has been well recognized that TEVAR should be considered for complicated hyperacute, acute, and subacute TBAD patients and for uncomplicated hyperacute, acute, and subacute TBAD patients with high-risk characteristics (18, 19). When the distance between the ostium of the LSA and the primary entry tear is <15 mm, or when the LSA is involved in the lesions, TEVAR with the Castor single-branched stent graft should be considered.

In addition, there are some reports on the use of the Castor single-branched stent graft in cases of zone 1 TEVAR: the LCCA is revascularized with the branch section, and the LSA is revascularized by creating an extra fenestration in the main body. This strategy is off-label and lacks verification, and should therefore be considered with great caution.

### 3.3. Evaluation of the anatomy

Computed tomography angiography (CTA) images and a workstation with centerline luminal reconstruction are necessary for precise CTA evaluation. The diameters of the LSA, true lumen, false lumen, and estimated true lumen at the estimated proximal and distal landing zone are measured to determine the proximal diameter of the stent graft, its taper ratio, and the length of the main body. The distance between the ostium of the LCCA and the LSA at the side of greater curvature is measured to determine the distance between the branch section and the proximal edge of the main body. The diameter of the LSA is measured to determine the diameter of the branch section. The distance between the ostium of the LSA and the ostium of the left vertebral artery (LVA) is measured to determine the length of the branch section, where coverage of the ostium of the LSA should be avoided. When the taper ratio of the main body cannot meet the requirements of the lesion, a distal-restrictive stent graft can be considered to avoid aortic rupture.

### 3.4. Oversize ratio

The suggested oversize ratio for aortic aneurysm is 20%; however, for aortic dissection, and especially for TEVAR with a single-branched stent graft, the oversize ratio should be reconsidered. An oversize ratio of 0%–5% for the proximal landing zone has been applied for endovascular repair of TBAD at many centers in China, which could greatly reduce the radial force of the stent graft on the fragile aortic wall. For a straight stent graft, the radial force is the main source of fixation. In the Castor single-branched stent graft, the unibody design allows the branch section to function as an anchor, thus providing extra strength for fixation. The fixation abilities of a straight stent graft and the Castor single-branched stent graft were compared *in vitro*, and it was found that a force of 24N was required in order for the Castor single-branched stent graft to migrate, which was twice that required in the case of the straight stent graft. Since the branch section could serve as an anchor to ensure fixation, the radial force generated from the oversized stent graft compressing the aortic wall could be reduced. Thus, an oversize ratio of 0%–5% for the proximal landing zone is acceptable for the prevention of migration, while ensuring tight adherence to the aortic wall to avoid type Ia endoleak.

## 4. Standard procedure for TEVAR with single-branched stent graft

### 4.1. Vascular access

The femoral artery (left or right) is the recommended conduit for delivery of the stent graft, and the left brachial artery (LBA) is the recommended conduit for access to the left subclavian artery. Once the LBA is cannulated, a catheter is advanced through the guide wire from the LBA to the exposed femoral artery to build the conduit for the branch section. After withdrawal of the guide wire within the conduit, the traction of the branch section is inserted into the conduit (catheter) from the femoral artery and acquired from the LBA.

### 4.2. Initial angiography

An extra-stiff guide wire (Lunderquist, Cook Medical, Bloomington, IN, USA) pigtail catheter is advanced into the ascending aorta from the femoral artery, and a pigtail catheter is advanced through the extra-stiff guide wire into the aortic arch. After withdrawal of the extra-stiff guide wire, the C-arm machine is set at the desired angle according to the preoperative measurement, such that all branches of the aortic arch can be viewed clearly, mostly between the left anterior oblique 30° and 60°. After these preparatory procedures, the initial angiography is performed to evaluate the morphological characteristics of the aorta and lesions.

### 4.3. Deployment of the main body

**Figure 1:**
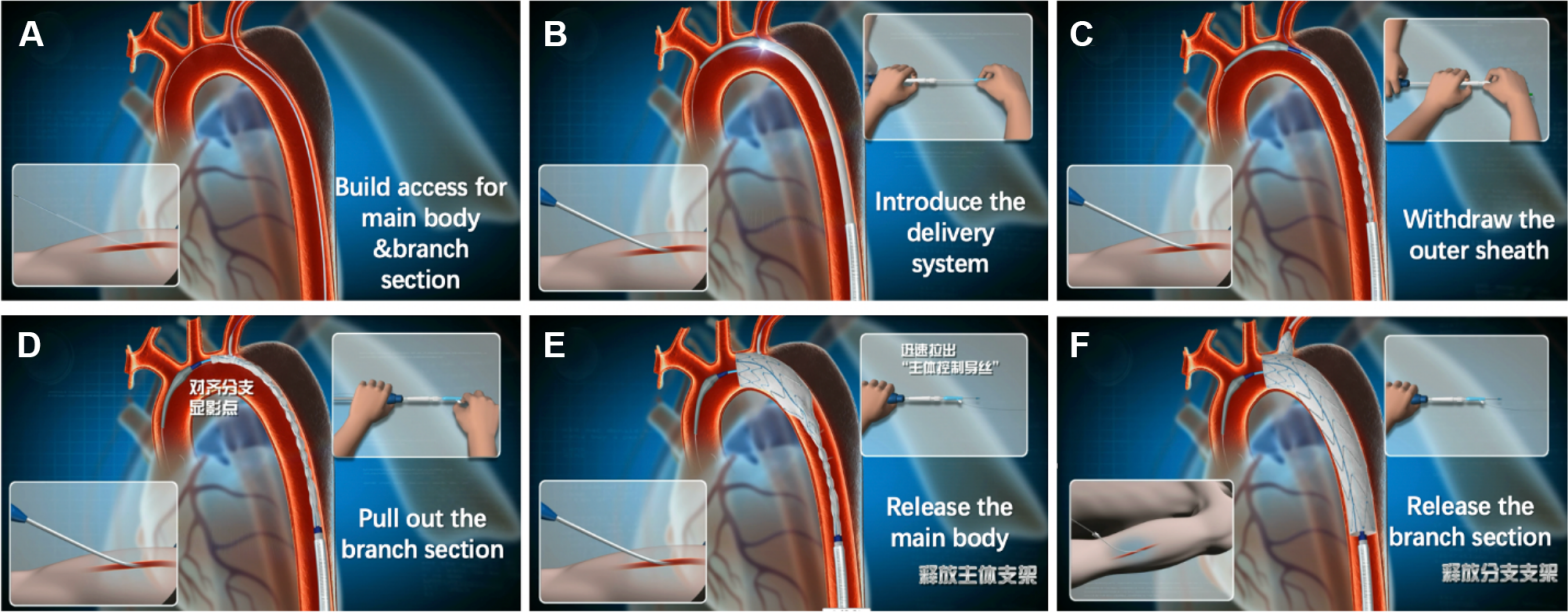
Deployment of the Castor single-branched stent graft. (**A**) Access for the main body and branch section; (**B**) introduction of the delivery system into the aortic arch; (**C**) withdrawal of the outer sheath in the thoracic descending aorta; (**D**) traction of the branch section; (**E**) release of the main body; (**F**) release of the branch section.

When angiography is complete, the extra-stiff guide wire should be inserted into the ascending aorta again and the pigtail catheter removed. The single-branched stent graft is then advanced over the extra-stiff guide wire into the thoracic descending aorta with the traction wire of the branch section simultaneously drawn at the point of the LBA. The outer sheath is removed and retained in the thoracic descending aorta, and the inner sheath (which is softer than the outer sheath), together with the stent graft, should be advanced into the aortic arch. After arrival at the target position, the inner sheath is removed and the branch section is dragged into the LSA by pulling the traction wire of the branch section. The main body of the single-branched stent graft is deployed by withdrawing the trigger wire, and the branched section is subsequently deployed by withdrawing the traction wire. After deployment of the single-branched stent graft, further angiography should be performed to verify complete exclusion of the lesion without endoleak, and to confirm that the LSA is patent (Figure 1).

### 4.4. Technical issues and details during deployment

Use of the direction inversion technique during deployment could lower the risk of misalignment between the branch section and the LSA. The clock position of the ostium of the LSA is within the range of 11:00–1:00 o’clock. When entering the femoral artery, the direction of the branch section should be pointed toward the floor, where the branch section will turn toward the direction of the ostium of the LSA at the aortic arch.

During advancement of the stent graft, rotation of the delivery system should be avoided. Even when no rotation occurs, there is a possibility that the traction wire of the branch section could become wrapped around the trigger wire of the main body. Therefore, before withdrawing the outer sheath, the operator should reconfirm that the traction wire and trigger wire are separated, or the operator should unwrap the guidewire by gently rotating the delivery system.

Although the branch section can be rotated by up to 150° in multiple directions while remaining patent, the operator should avoid rotation and misalignment of the branch section and the LSA through precise preoperative measurement and careful operation. When stenosis or twisting of the branch section occurs, this can be noted using angiography. In this situation, post-dilation should be considered as a solution.

## 5. Follow-up

Antiplatelet therapy should be considered in cases where the diameter of the branch section is <8 mm in order to prevent stenosis and occlusion of the branch section. Life-long surveillance is required after TEVAR with a single-branched stent graft. Contrast-enhanced CT is recommended at 1 month, 6 months, and 12 months and then annually after TEVAR for TBAD. For patients with contraindications to iodinated contrast agents, duplex ultrasound and non-contrast CT scan are recommended at 1 month, 6 months, and 12 months and then annually after TEVAR for TBAD.

## 6. Conclusion

An off-the-shelf single-branched stent graft simplifies the procedure for TEVAR with the requirement for LSA revascularization. With a steadier and shorter learning curve and a high success rate, the Castor single-branched stent graft is expected to become an important off-the-shelf option for endovascular repair of TBAD with insufficient healthy landing zone.

## Data Availability

The original contributions presented in the study are included in the article/Supplementary Material; further inquiries can be directed to the corresponding author.
